# Maternal hormonal milieu influence on fetal brain development

**DOI:** 10.1002/brb3.920

**Published:** 2018-01-24

**Authors:** Alexandra Miranda, Nuno Sousa

**Affiliations:** ^1^ Life and Health Sciences Research Institute (ICVS) School of Medicine University of Minho Braga Portugal; ^2^ ICVS/3B's ‐ PT Government Associate Laboratory Braga/Guimarães Portugal; ^3^ Department of Obstetrics and Gynecology Hospital de Braga Braga Portugal; ^4^ Clinic Academic Center ‐ 2CA Braga Portugal

**Keywords:** fetal neurodevelopment, fetal programming, glucocorticoids, maternal hormones, melatonin, oxytocin, sex steroids, thyroid hormones

## Abstract

An adverse maternal hormonal environment during pregnancy can be associated with abnormal brain growth. Subtle changes in fetal brain development have been observed even for maternal hormone levels within the currently accepted physiologic ranges. In this review, we provide an update of the research data on maternal hormonal impact on fetal neurodevelopment, giving particular emphasis to thyroid hormones and glucocorticoids. Thyroid hormones are required for normal brain development. Despite serum TSH appearing to be the most accurate indicator of thyroid function in pregnancy, maternal serum free T4 levels in the first trimester of pregnancy are the major determinant of postnatal psychomotor development. Even a transient period of maternal hypothyroxinemia at the beginning of neurogenesis can confer a higher risk of expressive language and nonverbal cognitive delays in offspring. Nevertheless, most recent clinical guidelines advocate for targeted high‐risk case finding during first trimester of pregnancy despite universal thyroid function screening. Corticosteroids are determinant in suppressing cell proliferation and stimulating terminal differentiation, a fundamental switch for the maturation of fetal organs. Not surprisingly, intrauterine exposure to stress or high levels of glucocorticoids, endogenous or synthetic, has a molecular and structural impact on brain development and appears to impair cognition and increase anxiety and reactivity to stress. Limbic regions, such as hippocampus and amygdala, are particularly sensitive. Repeated doses of prenatal corticosteroids seem to have short‐term benefits of less respiratory distress and fewer serious health problems in offspring. Nevertheless, neurodevelopmental growth in later childhood and adulthood needs further clarification. Future studies should address the relevance of monitoring the level of thyroid hormones and corticosteroids during pregnancy in the risk stratification for impaired postnatal neurodevelopment.

## 1 INTRODUCTION

In intrauterine life, mild and transient changes in maternal hormone levels, even within the currently accepted physiologic levels, can directly affect target gene expression profiles, which are generally involved in normal brain growth and maturation (Brunton & Russell, 2011; Morreale de Escobar et al., 2004b). Importantly, hormone effects on brain development are found to be time‐ and dose‐dependent, with exposure to abnormal levels outside the critical period having limited impact (Auyeung, Lombardo, & Baron‐Cohen, 2013). Some fetal hormonal axes are particularly susceptible to long‐term programming effects that can persist throughout life and result in impaired brain growth, altered behavior, and increased susceptibility to chronic disease (such as metabolic and psychiatric disease). Nevertheless, long‐term effects reflect an activation or fine‐tuning of the early organization of the brain. Epigenetic mechanisms may underlie such consequences that, in some cases, are only evident in subsequent generations (Auyeung et al., 2013; Cottrell & Seckl, 2009; Harris & Seckl, 2011). In this review, we will provide an update of the research data on maternal hormonal impact on fetal neurodevelopment, giving particular emphasis to thyroid hormones and glucocorticoids, for which the relevance for fetal neurodevelopment is well established, the body of published scientific evidence is robust, and clinical guidelines are already available for hormonal replacement in particular circumstances. In addition, there is a known cross talk between these two axes that has already started to be described and is thought to be of relevance in the womb. To facilitate the comprehension of the topic, the influence of each of these hormones will be discussed separately and a final integrative analysis will be provided. A final glimpse on the influence of maternal sex steroids, oxytocin, and melatonin on fetal neurodevelopment will also be given.

## 2 THYROID HORMONES

### 2.1 Thyroid hormone axis: Ontogeny, metabolism, and molecular signaling in the developing brain

Activation of the thyroid hormone axis follows the production of thyrotropin‐releasing hormone (TRH) at the hypothalamus and stimulation of thyrotropin (TSH) release from the pituitary. TSH in turn increases prohormone thyroxine (T4) production and, to a lesser extent, its active counterpart, tri‐iodothyronine (T3). Both T4 and T3 feedback to inhibit excessive TSH production (Fisher, Dussault, Sack, & Chopra, 1976). During the first half of pregnancy, maternal thyroid hormone production and iodine requirements increase. Total T4, free T4, and T4 binding globulin are expected to increase, mainly after week 7 of gestation, while TSH is expected to decrease because of the thyrotropic activity of elevated circulating human chorionic gonadotropin (hCG) concentrations. In the second and third trimesters, serum TSH gradually increases, but the TSH reference interval remains lower than in nonpregnant women (Haddow, Knight, Palomaki, McClain, & Pulkkinen, 2004; Stricker et al., 2007). Several studies reported gestational age‐specific reference intervals for maternal thyroid function tests (Lazarus et al., 2012; Springer, Bartos, & Zima, 2014; Vaidya et al., 2007; Yang et al., 2014). Serum TSH seems to be the most accurate indicator of thyroid status in pregnancy, despite substantial population differences in the TSH upper reference limit (Alexander et al., 2017; De Groot et al., 2012). Recent studies identified only a slight decrease in the upper reference range of TSH after week 7–12 of gestation. Additionally, a low but detectable TSH in the first trimester of pregnancy is likely not clinically significant, as increased levels of serum hCG directly stimulate thyroid hormone production and promote a negative feedback on TSH secretion (Alexander et al., 2017). The American Thyroid Association recommends using population‐based, trimester‐specific reference ranges for thyroid function determination in pregnancy (Alexander et al., 2017). Notwithstanding, TSH levels may differ widely, mainly in the first trimester of pregnancy, according to several factors such as women parity, iodine status, and body mass index (Laurberg, Andersen, Hindersson, Nohr, & Olsen, 2016). This will eventually lead to misclassification and, possibly, to incorrect therapeutic institution when using standardized reference ranges. The different thyroid function tests available for determination in pregnancy are shown in Table 1.

**Table 1:** Thyroid function tests in pregnancy

Free T4	Each laboratory should establish method and trimester‐specific reference ranges
	Optimal method: LC/MS/MS, although time‐consuming, expensive, and not widely available
	Automated immunoassays most widely used, but usually overestimate free T4 levels
Free T4 index (“adjusted T4”)	Appears to be reliable during pregnancy
	Total T4 × T3 resin uptake ratio (T3 ratio)
Total T4	May be superior to free T4 measurements in pregnant women
	Multiply the nonpregnant total T4 range (5–12 μg/dl or 50–150 nmol/L) by 1.5‐fold after week 16
	Between weeks 7–16 of pregnancy, the upper reference range is calculated by increasing the nonpregnant upper reference limit by 5% per week
TSH	More accurate indicator of thyroid status in pregnancy
	After week 7 of gestation, the lower reference range of TSH can be reduced ~0.4 mU/L and the upper reference range is reduced ~0.5 mU/L (TSH upper reference limit of 4.0 mU/L)
	Gradual return toward the nonpregnant range in the second and third trimesters

**Figure 1:**
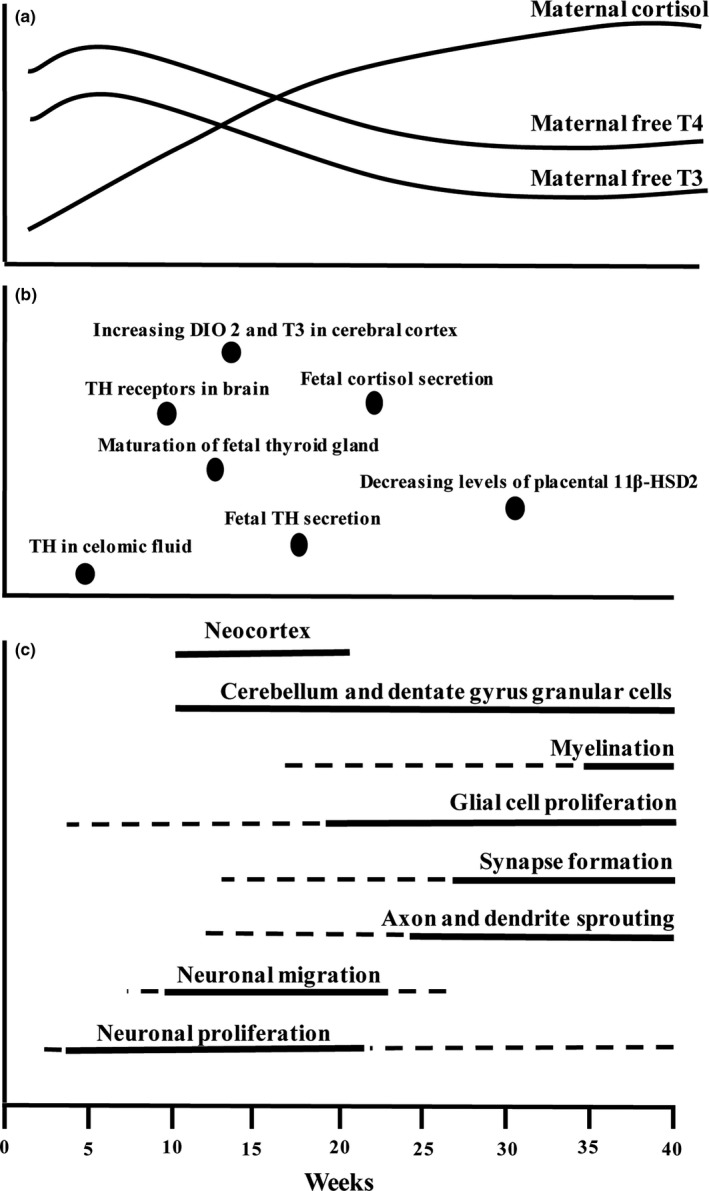
(a) The relative concentrations of maternal cortisol and free thyroid hormones during pregnancy; (b) important time points in the ontogeny of fetal cortisol and thyroid hormone function and metabolism; (c) time‐specific actions of HPA and HPT axes on fetal brain development. Figure adapted from Patel et al., 2011

**Figure 2:**
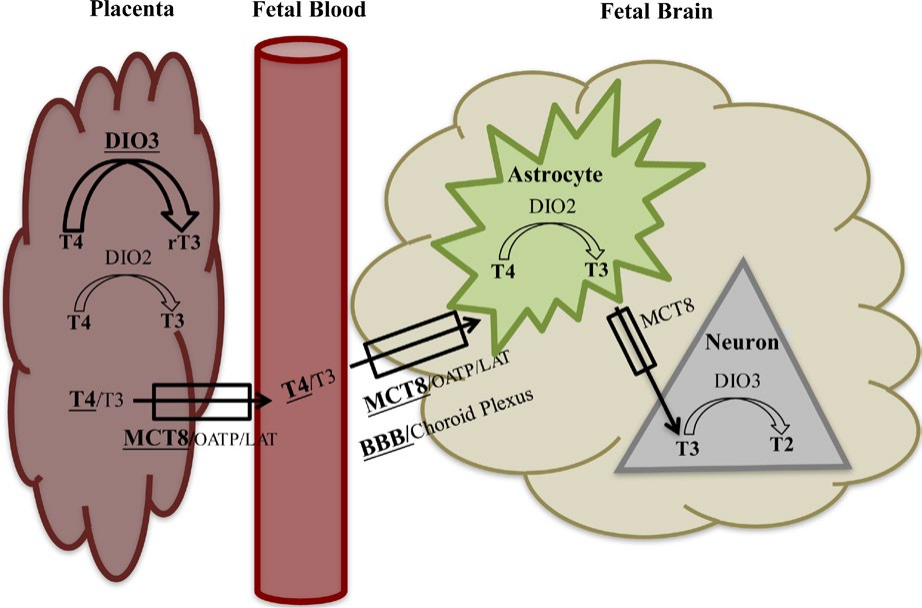
Thyroid hormone transport and metabolism between placenta and fetal brain in humans. DIO3 activity in placenta is up to 400 times greater than that of DIO2, and the most relevant thyroid hormone membrane transporter in humans is MCT8 transporter. Thyroid hormones are delivered to the brain mainly through the blood–brain barrier (BBB) with a smaller fraction (about 20%) being transported through the choroid plexus. Fetal brain is mainly dependent on circulating T4 levels, with 80% of T3 in the cerebral cortex being produced by local deiodination of free T4 in astrocytes. DIO, deiodinase; BBB, blood–brain barrier

Critical amounts of maternal T3 and T4 must be supplied across the placenta to the fetus to ensure the correct neurodevelopment throughout ontogeny. Detection of T4 and T3 in the human cerebral cortex has been described by week 12 of gestation (Figure 1; Morreale de Escobar et al., 2004b; Morreale de Escobar, Obregón, & Escobar del Rey, 2007). Thyroid hormone transport and metabolism were shown to be important for thyroid hormone availability and actions in the fetal brain (Landers & Richard, 2017). In humans, thyroid hormones are delivered to the brain mainly through cerebral circulation (blood–brain barrier), with a smaller fraction (about 20%) being transported through the choroid plexus (Bernal, 2015). Several thyroid hormone membrane transporters have been described; however, the most relevant are monocarboxylate transporter 8 (Mct8), with affinity for both T3 and T4, and organic anion transporter 1C1 (OATP1C1), which has much higher selectivity for T4 (Landers & Richard, 2017; Figure 2). In fact, developing human brain is relatively impermeable to T3, with about 80% of T3 in the cerebral cortex being produced by local deiodination of free T4 (Faustino & Ortiga‐Carvalho, 2014; Guadaño‐Ferraz, Obregón, St Germain, & Bernal, 1997; Figure 2). Briefly, after being taken up by astrocytes, T4 is deionized to T3 by deiodinase type 2 and, posteriorly, T3 is exported through MCT8 transporter (Guadaño‐Ferraz et al., 1997; Roti, Fang, Green, Emerson, & Braverman, 1981). Oligodendrocytes and neurons use the same transporter for uptake of T3 (Heuer et al., 2005). Deiodinase type 3, highly expressed in neurons, is positively regulated by thyroid hormone and protects different brain regions from untimely or excessive T3 levels, by converting T4 to the biologically inactive reverse T3 (Crantz, Silva, & Larsen, 1982; Landers & Richard, 2017; Figure 2).

All these findings emphasize the need for maternal T4 levels to be maintained within strict levels to ensure normal fetal brain development until maturation of fetal thyroid gland (by week 11–12) and thyroid hormone secretion (around week 16); (Obregon, De Oña, Calvo, Escobar del Rey, & Morreale de Escobar, 1991). Even after onset of fetal thyroid secretion, human data show that maternal transfer still represents about 30%–60% of fetal serum T4 and continues to have an important protective role in fetal neurodevelopment until birth (Morreale de Escobar et al., 2004b; Vulsma, Gons, & de Vijlder, 1989).

The majority of thyroid hormone actions in brain development are mediated through the binding of T3 to nuclear thyroid hormone receptors, which are highly expressed in oligodendrocytes and neurons, and act as transcription factors that modulate the expression of target genes (Bradley, Towle, & Young, 1992). There are two thyroid receptor genes encoding three proteins with full thyroid hormone receptor function at the genomic level (Iskaros et al., 2000). Despite isoform‐specific roles, their main actions largely overlap. Thyroid hormone receptors are already expressed in human brain by the 10th week of gestation, with a several fold increase in expression within the following weeks (Figure 1). There is also a distinct spatial expression pattern of each isoform during cortex development, with prominent expression in cerebellum and in the germinal trigone and cortical ventricular layer, where neuroblast proliferation takes place (Bradley, Young, & Weinberger, 1989; Bradley et al., 1992; Chatonnet, Picou, Fauquier, & Flamant, 2011). Recent evidence has also described nongenomic action of thyroid hormones via integrin a_v_b_3_, a cell membrane receptor (Stenzel & Huttner, 2013).

The molecular mechanisms by which thyroid hormones affect fetal neurological structures are still not fully understood. In neural cells, it is estimated that around 5% of all expressed genes are under T3 control, mostly by direct regulation at transcriptional level (Gil‐Ibanez, Bernal, & Morte, 2014). Nevertheless, influence of thyroid hormones in gene regulation can also be accomplished at post‐transcriptional level, by affecting proteins involved in RNA stability and splicing (Aniello, Couchie, Bridoux, Gripois, & Nunez, 1991; Bernal, 2015).

Thyroid hormone is required early in pregnancy for normal neurogenesis, neuronal migration, neuronal and glial cell differentiation, myelination, and synaptogenesis (Bernal, 2017; Eayrs, 1953; Lavado‐Autric et al., 2003). In fact, thyroid hormones were shown to modify expression of genes associated with cell cycle and intracellular signaling (E2F1, p53, cyclins, and cyclin‐dependent kinase inhibitors), cytoskeleton organization (actin and microtubule polymerization), and extracellular matrix contents (laminin, fibronectin, and several adhesion molecules), which are involved in neurogenesis and neuronal migration (Bernal, 2017; Farwell & Dubord‐Tomasetti, 1999; Iglesias et al., 1996; Koibuchi, Jingu, Iwasaki, & Chin, 2003; Leonard & Farwell, 1997; Lin et al., 2004;). Neurogenin 2 and Reelin are also proteins involved in neuron development and neuronal migration which expression has also been proven to be modulated by thyroid hormones (Dong et al., 2009; Pathak, Sinha, Mohan, Mitra, & Godbole, 2011). In animal and cell culture models, thyroid hormone treatment after traumatic brain injury or hypoxic event significantly increased, mainly on cortex, the expression of mRNA from Bcl2, VEGFA, Sox2, and neurotropin, which are important genes for neuronal survival and neurogenesis (Li et al., 2017). Thyroid hormone can also influence oligodendrocyte differentiation, which has an important impact on the process of myelination (Schoonover et al., 2004). Indeed, thyroid hormone affects the expression of almost all myelin protein genes, particularly those encoding the structural proteins (proteolipid protein, myelin basic protein and myelin associated glycoprotein); (Sutcliffe, 1988). Additionally, thyroid hormones lead to changes in the expression of nuclear‐encoded mitochondrial cytochrome c oxidase subunits and mitochondrial‐encoded mRNAs, such as 12S and 16S RNAs, thereby affecting mitochondrial morphology and function, namely, electron transport chain processing (Vega‐Núñez, Menéndez‐Hurtado, Garesse, Santos, & Perez‐Castillo, 1995).

### 2.2 Maternal thyroid dysfunction and fetal and postnatal neurodevelopment

The role of thyroid hormone in postnatal brain development has been extensively studied, and this effort resulted in the prevention, by neonatal screening, of severe neurological deficits, including profound neurological impairment and mental retardation, in children with congenital hypothyroidism (Delange, 1997). The importance of thyroid hormone to fetal brain development has been less characterized, but it is well established that maternal thyroid status is also intricately involved in the developing fetal brain (Eayrs, 1953; Lavado‐Autric et al., 2003; Patel, Landers, Li, Mortimer, & Richard, 2011; Stenzel & Huttner, 2013). Maternal thyroid dysfunction generally ensues in response to several factors such as iodine deficiency, environmental endocrine disrupters, or intrinsic thyroid diseases (Min et al., 2015). World Health Organization (WHO) has declared that iodine deficiency is, after starvation, the single most important cause of preventable brain damage, by affecting thyroid function (Morreale de Escobar et al., 2007; WHO, 2007). Although severe iodine deficiency almost no longer exists, due to widespread programs of dietary iodine supplementation (in bread, milk, water, or salt), mild iodine deficiency is still a public health concern, either in developing countries and in Western industrialized nations, with Europe having the highest proportion (almost 57%) of general population with an insufficient iodine intake (World Health Organization/International Council for the Control of the Iodine Deficiency Disorders/United Nations Children's Fund (WHO/ICCIDD/UNICEF), 2007). The most vulnerable groups are pregnant and lactating women and their developing fetuses and neonates, given the outstanding importance of iodine to ensure adequate levels of thyroid hormones for brain development and maturation.

Increasing body of evidence suggests that a “fetal programming” effect, which explains the association between environmental adversity during pregnancy and later development of disease, may drive offspring of women with thyroid dysfunction susceptible to later onset of neurodevelopmental disorders (Andersen, Carlé, Karmisholt, Pedersen, & Andersen, 2017; Barker, Winter, Osmond, Margetts, & Simmonds, 1989). Unfortunately, all human studies addressing the association between maternal thyroid status in pregnancy and child neurodevelopment are observational and challenged by some methodological concerns. It is worth noting the heterogeneity between studies regarding thresholds used to define thyroid dysfunction subgroups, gestational age at assessment, length of follow‐up period, and tools for the assessment of child neurodevelopment. Due to ethical issues, randomized controlled trials are few and have been conducted only with child IQ (Casey, 2016; Lazarus, 2010; Lazarus et al., 2012).

In humans, the most severe and adverse effect of maternal thyroid dysfunction on fetal brain development is the neurological symptoms of endemic cretinism associated with severe maternal hypothyroidism (Chen & Hetzel, 2010). Nevertheless, several other neurodevelopmental deficits have been described for more moderate forms of maternal thyroid dysfunction, particularly during the first half of pregnancy (Henrichs, Ghassabian, Peeters, & Tiemeier, 2013; Moog et al., 2017).

In large Danish population‐based studies, maternal thyroid dysfunction first time diagnosed after birth was associated with a significant risk of epilepsy during childhood (Andersen, Laurberg, Wu, & Olsen, 2013). The same authors concluded that maternal hyperthyroidism diagnosed after the birth of the child increased the risk of attention‐deficit/hyperactivity disorder during childhood, whereas hypothyroidism increased the risk of autism spectrum disorder (Andersen, Laurberg, Wu, & Olsen, 2014).

Additionally, two early observational studies demonstrated that offspring of women with asymptomatic thyroid dysfunction were at increased risk of impaired neurodevelopment (Hollowell, Garbe, & Miller, 1999; Pop et al., 1999). Particularly, Hollowell et al. (1999), in a large case–control study, demonstrated that offspring of untreated hypothyroid women, at 7–9 years of age, performed worse on all the 15 neuropsychological tests assessing intelligence, attention, language, reading ability, school performance, and visual–motor performance. The detrimental effect of subclinical hypothyroidism on fetal neurocognitive development is less clear. Nevertheless, maternal hypothyroxinemia, frequently due to mild iodine deficiency, is one leading cause of preventable neurodevelopmental handicaps. Hypothyroxinemia consists of low T4 levels in maternal serum (generally defined as total T4 levels below 100 nmol/L and free T4 concentrations in the lower 2.5th–5th percentile of the reference range), with T3 and TSH levels within normal range (Alexander et al., 2017). Briefly, insufficient iodine intake promotes increased synthesis of T3, instead of T4, leading to reduced iodine demand, while keeping TSH levels within normal range. Although this adaptation is advantageous for the mother, maternal thyroid hormone supply to the fetus can be compromised as T4 is the main thyroid hormone crossing the placenta and entering the fetal brain. Maternal hypothyroxinemia represents a paradigm of how an innocuous and asymptomatic endocrine maternal status, hardly diagnosed in clinical practice, can have a lifetime impact on the fetus intellectual performance.

Costeira et al. (2010) found an incidence of isolated hypothyroxinemia of almost 2.6% in a population of Portuguese pregnant women reported to be iodine‐deficient. Pop et al. (2003) described that the strongest isolated predictor of infant mental development was mother's free T4 levels at 12 weeks of gestation, and the lowest developmental scores were observed in the children of mothers whose free T4 levels were low in the first trimester and decreased further over pregnancy. Similarly, Costeira et al. assessed the psychomotor development of the progeny of women from a moderately iodine‐deficient area and found that maternal serum free T4 levels in the first trimester of pregnancy were the major determinant of psychomotor development at 18 and 24 months. Children born from mothers with free T4 levels <25th percentile (<10 pg/ml), which represented approximately 25% of the sample, had an odds ratio of 2.1 for mild‐to‐severe delay (Costeira et al., 2011). It is important to highlight that, despite increased risk of children with developmental delay, a considerable amount of these women had free T4 levels within the currently accepted physiologic range. Human studies performed only in the second half of pregnancy have found no association between maternal hypothyroxinemia and children cognitive outcomes until 60 months of age, as well as language and motor scores at age 24 months (Chevrier et al., 2011; Craig et al., 2012).

The Generation R Study, involving 3,659 children and their mothers, reported a neurodevelopmental index more than one standard deviation below the mean in one of every two offspring from women with first trimester free T4 below the 10th percentile (1 in 20 births), resulting in a 1.5‐fold to twofold increased risk of adverse cognitive findings at 3 years of age (Henrichs et al., 2010). In this study, both mild and severe maternal hypothyroxinemia were associated with a higher risk of expressive language delay, and severe maternal hypothyroxinemia also predicted a higher risk of nonverbal cognitive delay. These findings are supported by previous studies showing similar relationships (Li et al., 2010; Pop et al., 2003; Velasco et al., 2009). Two other prospective studies, performed within the Generation R birth cohort in the Netherlands, investigated the relationship between maternal thyroid function and children IQ at 6 years, as well as different brain structural changes at 8 years. One study reported that children of mothers that had been hypothyroxinemic in early pregnancy scored 4.3 points IQ lower than controls without differences in brain morphology (Ghassabian et al., 2014). The other study, within the same cohort, found that, after the exclusion of women with overt hypothyroidism and overt hyperthyroidism, both low and high maternal free thyroxine concentrations during pregnancy were associated with lower child IQ and lower gray matter and cortex volume (Korevaar et al., 2016). Maternal TSH was not related to the cognitive outcomes nor brain morphology. Brain structural changes were also described for more pronounced levels of maternal thyroid dysfunction. Particularly, small studies from Canada reported reduced hippocampal volumes, abnormal development of corpus callosum, and abnormal cortical morphology in children aged 9–14 years born to women who were treated with levothyroxine during pregnancy for previously or newly detected hypothyroidism (Lischinsky, Skocic, Clairman, & Rovet,^2016^; Samadi, Skocic, & Rovet, 2015; Willoughby, McAndrews, & Rovet, 2014).

The neuroanatomical and functional consequences of maternal thyroid dysfunction during early development have been characterized in animal models. As an example, in rodents, maternal thyroid hormone deficiency resulted in a reduced neural progenitor pool in the ventricular zone (Mohan et al., 2012). The abnormal neurogenesis may be attributed, at least in part, to the oxidative damage and deteriorated antioxidant defense system induced by the hypothyroid state (Ahmed, Ahmed, El‐Gareib, El‐Bakry, & Abd El‐Tawab, 2012). Maternal hypothyroidism during pregnancy also limited dendritic and axonal growth, and induced abnormal neuronal location and synaptic impairment (Farwell, Dubord‐Tomasetti, Pietrzykowski, Stachelek, & Leonard, 2005; Opazo et al., 2008). Even a moderate transient period of maternal hypothyroxinemia at the beginning of rat neurogenesis can promote an abnormal neuronal migration pattern, causing alterations in histogenesis and cytoarchitecture of the somatosensory cortex, hippocampus, and cerebellum that could be prevented by timely infusion of T4. Delayed infusion beyond the critical period of corticogenesis was of no benefit (Lavado‐Autric et al., 2003). In fact, third trimester or early postnatal correction of the low circulating T4, which is usually effective in preventing most neurological damage in congenital hypothyroidism, does not reverse the structural deficits caused by maternal hypothyroxinemia because most of the consequences have become permanent by the end of the second trimester (Morreale de Escobar, Obregón, & Escobar del Rey, 2004a; Morreale de Escobar et al., 2004b; Zoeller, 2003). To what extent must maternal T4 levels decline before effects on cortical structure are observed and what measures of thyroid function should be taken to best determine the health of the fetus are still unanswered questions. These structural abnormalities might have a postnatal functional impact. Indeed, hypothyroidism during the fetal period has been associated with the impairment of spatial learning and memory in rat offspring (Shafiee, Vafaei, & Rashidy‐Pour, 2016). Additionally, subcortical band heterotopia, a class of neuronal migration error, was associated with increased sensitivity to seizures in offspring of female rats exposed to a goitrogen during pregnancy (Gilbert, Ramos, McCloskey, & Goodman, 2014).

### 2.3 Treatment options and guidelines

Thyroid dysfunction in pregnancy is a relevant issue as thyroid disease is the second most common endocrine disorder affecting women at reproductive age (ACOG, 2002). Notwithstanding, screening pregnant women for thyroid dysfunction remains controversial (Costeira et al., 2010; Granfors et al., 2014). The American Thyroid Association guidelines recommended a case finding–screening strategy in pregnancy (Alexander et al., 2017; Table 2). However, the Endocrine Society Committee for 2012 guidelines could not reach agreement with regard to screening recommendations for all newly pregnant women (De Groot et al., 2012). Some members advocated for routine TSH determination during the first trimester of pregnancy, whereas others, with a lower grade of evidence, supported aggressive case finding to identify and test high‐risk women.

**Table 2:** Risk factors for thyroid dysfunction in pregnancy

Age > 30 years
Family history of autoimmune thyroid disease or thyroid dysfunction
History of hypothyroidism/hyperthyroidism or current symptoms/signs of thyroid dysfunction
Known thyroid antibody positivity or the presence of a goiter
History of head or neck radiation or prior thyroid surgery
Personal history type 1 DM or other autoimmune disorders
History of pregnancy loss, preterm delivery, or infertility
Multiple prior pregnancies (≥2)
Morbid obesity (BMI ≥ 40 kg/m^2^)
Use of amiodarone or lithium, or recent administration of iodinated radiologic contrast
Residing in an area of known moderate to severe iodine insufficiency

In fact, despite the absence of large randomized trials demonstrating the benefit of routine screening of thyroid dysfunction during pregnancy, according to Vaidya et al. (2007), an aggressive case finding approach is known to miss about one‐third of women with overt and subclinical thyroid disease. Several authors share similar results (Casey & de Veciana, 2014; Li et al., 2010; Negro et al., 2010; Wang et al., 2011).

The critical question is whether thyroid replacement would benefit pregnant women identified with milder thyroid dysfunction and their offspring, particularly concerning children neurological development. Some observational studies have suggested the benefit of treatment (Li et al., 2010; Negro et al., 2006), but conversely, the larger Controlled Antenatal Thyroid Screening (CATS) randomized controlled trial demonstrated that thyroxine replacement in women with isolated high TSH or isolated low free T4 levels had no impact on cognitive function in children evaluated at 3 years old (Lazarus, 2010; Lazarus et al., 2012). Another multicenter randomized placebo‐controlled clinical trial, the Randomized Trial of Thyroxine Therapy for Subclinical Hypothyroidism or Hypothyroxinemia Diagnosed During Pregnancy, selected 677 women with subclinical hypothyroidism and 526 women with isolated maternal hypothyroxinemia to T4 treatment or placebo, showing no significant effect of treatment on offspring IQ at the age of 5 years (Casey, 2016). Therefore, these results provide evidence against treatment of subclinical hypothyroidism and hypothyroxinemia to improve neurocognitive outcomes in offspring. Nevertheless, both studies initiated T4 replacement at the end of the first trimester or later, which may be too late to have a significant impact on neurodevelopment. Two randomized trials evaluating the effect of T4 replacement upon fetal neurodevelopment are currently in progress. Meanwhile, according to American Thyroid Association guidelines, T4 replacement is recommended for women with overt and subclinical hypothyroidism. Thyroid hormone replacement with T3 or T4 + T3 combinations should be avoided for the treatment of maternal hypothyroidism during pregnancy (Alexander et al., 2017). In comparison with human thyroid hormone concentrations, those preparations have supraphysiologic levels of T3 that can lead not only to maternal adverse effects but also to an insufficient transfer of maternal T4 to the fetal brain. Despite management of maternal hypothyroxinemia being controversial and requiring further study, the panel suggests this condition should not be routinely treated in pregnancy (weak recommendation); (Alexander et al., 2017). In fact, it should be kept in mind that, until more consistent evidence is available on the impact of maternal hyperthyroidism on fetal neurodevelopment, the advisability of T4 replacement in cases of milder maternal thyroid hormone deficiency, such as hypothyroxinemia, should be cautious.

Concerning the high prevalence of iodine‐deficient intake worldwide, several health societies recommend that all women who are planning to be pregnant, are pregnant or breastfeeding, should supplement their diet daily with an oral supplement containing 150 μg of iodine (Alexander et al., 2017; De Groot et al., 2012; World Health Organization/International Council for the Control of the Iodine Deficiency Disorders/United Nations Children's Fund (WHO/ICCIDD/UNICEF), 2007).

In conclusion, identification of maternal thyroid dysfunction during pregnancy is particularly important in the first trimester when fetal thyroid hormones rely exclusively on the mother. It negatively impacts on the psychomotor development and intelligence coefficient of the offspring, even for TSH levels within range for pregnancy. Nevertheless, many clinicians and researchers agree that establishing reference ranges for thyroid hormones during pregnancy is required for proper monitoring (Table 3). In contrast to hypothyroid spectrum disorders (overt hypothyroidism, subclinical hypothyroidism, and hypothyroxinemia), studies assessing the relationship between maternal hyperthyroidism and fetal neurodevelopmental outcomes are still limited.

**Table 3:** Thyroid hormones: Issues on debate

Understand the molecular mechanisms by which thyroid hormones affect fetal neurodevelopment
Determine reference ranges for thyroid hormones during pregnancy and the most informative parameters
Clarify the detrimental effect of maternal subclinical hypothyroidism and hyperthyroidism on fetal neurocognitive development
Establish cutoff points of free T4 to define hypothyroxinemia and the levels of maternal T4 below which negative effects on brain structure are observed
Elucidate the impact of routine screening of thyroid dysfunction and hormone replacement of pregnant women with milder thyroid dysfunction on their offspring neurodevelopment outcome

## 3 GLUCOCORTICOIDS

### 3.1 Hypothalamus–pituitary–adrenal axis: ontogeny, metabolism, and molecular signaling in the developing brain

Glucocorticoids are steroid hormones with an essential role in fetal development and maturation, as well as in adult homeostasis (Liggins, 1976). The limbic system, mainly through the hippocampus, sends an inhibitory input to the paraventricular nucleus (PVN) of the hypothalamus, by activation of GABAergic neurons. This results in increased production and release of corticotropin‐releasing hormone (CRH) and, to a lesser extent, arginine vasopressin (AVP), which act on the anterior pituitary to stimulate adrenocorticotropic hormone (ACTH) production and release (Edwards & Burnham, 2001). In response, there is an increase in glucocorticoid release by the adrenal glands that feedback at the level of the hippocampus, hypothalamus, and pituitary to inhibit HPA axis activity and prevent excessive production of stress hormones (Cottrell & Seckl, 2009). The human fetal adrenal gland is active from very early gestation onward, but adrenal cortisol is only produced in appreciable quantities after 22 weeks of gestation (Liggins, Kennedy, & Holm, 1967; Weinstock, Matlina, Maor, Rosen, & McEwen, 1992); (Figure 1). Thus, in early gestation, cortisol of maternal origin represents the primary source of cortisol and correlates strongly with fetal compartment levels, with cortisol increasing over the three trimesters (Harris & Seckl, 2011); (Figure 1).

During pregnancy, several changes in maternal and fetal HPA axis are observed. Different studies have focused on the role of cortisol in the coordination of fetal readiness for extrauterine life and the timing of parturition (Liggins et al., 1967; McDonald & Nathanielsz, 1991; Wood & Keller‐Wood, 2016). Human placenta produces CRH and ACTH that are delivered into fetal and maternal circulation (Goland et al., 1993). Placental CRH production increases over gestation and, in contrast to the negative feedback actions of glucocorticoids on central CRH expression, placental production of CRH is enhanced by glucocorticoids, creating a positive feedback loop that enhances maternal and fetal CRH, ACTH, and cortisol production, mainly in the third trimester of pregnancy (King, Smith, & Nicholson, 2001; Marinoni, Korebrits, Di Iorio, Cosmi, & Challis, 1998; Robinson, Emanuel, Frim, & Majzoub, 1988). In fact, maternal cortisol levels increase almost fourfold during pregnancy (Davis & Sandman, 2010). Corticosteroids act by suppressing cell proliferation and DNA replication and stimulating terminal differentiation (Liggins, 1976). These effects are of utmost importance in late gestation for the maturation of fetal organ systems, such as lung, liver, skeletal muscle, kidney, and central nervous system (Ballard & Ballard, 1972; Liggins, 1976; Wood & Keller‐Wood, 2016). Nevertheless, these maturation processes are performed at the expense of general slowing of somatic growth (Fowden & Forhead, 2011; McDonald & Nathanielsz, 1991).

Particularly, the developing brain is extremely sensitive to neurochemical, structural and molecular corticosteroid stress hormone effects (Meyer, 1983; Yehuda, Fairman, & Meyer, 1989). The ontogeny of corticosteroid receptors in the brain and the development of feedback control have been studied primarily in rats (Edwards & Burnham, 2001). Corticosteroids reach the brain and interact with two types of receptors, the mineralocorticoid receptors (MR) and the glucocorticoid receptors (GR) which, upon ligand binding in the cytoplasm, act as transcription factors in the nucleus, either by direct interaction with DNA recognition sites (glucocorticoid response elements) or through interaction with other transcription factors, in the promoter region of target genes (Groeneweg, Karst, de Kloet, & Joëls, 2011; Reul & de Kloet, 1985).

Although with an important overlap, MR and GR show different localization patterns in the brain and bind to different sets of genes, exhibiting a coordinated and often antagonistic mode of action (Edwards & Burnham, 2001). Particularly, MR are almost restricted to neurons in limbic areas, such as prefrontal cortex, amygdala, and, mainly, hippocampus (Groeneweg et al., 2011). In contrast, GR are widely distributed and highly expressed in placenta and brain, both in glia cells and neurons, with particular emphasis in the hippocampus, PVN, and pituitary corticotrophs (Diaz, Brown, & Seckl, 1998; Edwards & Burnham, 2001; Reul & de Kloet, 1985). In rats, MR start to be expressed in late gestation and are found at adult levels by the end of the first week of life, while GR are expressed from midgestation onward, reaching adult levels by about the first month of life (Diaz et al., 1998; Reul & de Kloet, 1985). A detailed analysis of the ontogeny of corticosteroid receptors expression in the human fetal brain has not been reported. MR have a 10‐fold higher affinity for circulating endogenous glucocorticoids than GR and are therefore responsible for the maintenance of basal HPA activity, while GR signal mainly during acute stress (Funder, 1997; Reul & de Kloet, 1985). With prolonged stress, chronic activation of GR promotes alteration in the number of hippocampal and hypothalamic GR, thereby modifying feedback regulation of the HPA axis (Towle, Sze, & Lauder, 1982). Importantly, although endogenous glucocorticoids can bind both GR and MR, synthetic glucocorticoids are more selective and bind almost exclusively to GR (De Kloet, Fitzsimons, Datson, Meijer, & Vreugdenhil, 2009; Joëls, Karst, DeRijk, & de Kloet, 2008).

Target genes of HPA axis include several functional classes of genes involved in energy metabolism, neuronal cell division and plasticity, cytoskeletal remolding, vesicle dynamics, and cell adhesion (Antonow‐Schlorke, Schwab, Li, & Nathanielsz, 2003; Datson, Morsink, Meijer, & de Kloet, 2008; Fukumoto, Morita, Mayanagi, & Tanokashira, 2009). Corticosteroids effects are often mediated by epigenetic changes in several genes, through DNA methylation and specific histone residue acetylation, and by synaptic plasticity (Murgatroyd et al., 2009; Weaver et al., 2007).

Besides delayed genomic effects of corticosteroids in the developing brain, mediated by transcriptional regulation, evidence also supports the presence of rapid nongenomic corticosteroid effects mediated by cell membrane receptors (Drake, Tang, & Nyirenda, 2007). Several studies have shown that membrane‐associated G protein‐coupled receptors and the signal‐regulated kinase–CREB pathway may be involved on the activation of neurons in limbic areas (Groeneweg et al., 2011). The same downstream signaling cascades also seem to be activated by other steroids, such as estrogens, androgens, and progesterone (Groeneweg et al., 2011; Levin, 2008).

Glucocorticoids metabolism is also of relevance concerning fetal exposure to these hormones. Human placenta expresses the enzyme 11β‐hydroxysteroid dehydrogenase types 1 and 2 (11β‐HSD1 and 11β‐HSD2), which interconvert cortisol and cortisone. As the predominant reaction is the placental inactivation of active glucocorticoids (cortisol) to their inactive 11‐keto forms (cortisone) by 11β‐HSD2, fetuses are usually protected from the relatively high maternal glucocorticoid levels during pregnancy (Liggins, 1976). Nevertheless, some of the cortisone synthesized during transplacental exchange is converted back to cortisol in target tissues, including brain (Wood & Keller‐Wood, 2016). During late gestation, 11β‐HSD2 placental expression decreases, exposing the fetus to increasing levels of glucocorticoids and facilitating fetal maturation (Cottrell, Seckl, Holmes, & Wyrwoll, 2014); (Figure 1). Importantly, synthetic glucocorticoids, such as dexamethasone and betamethasone, are not subject to substantial inactivation by placental 11β‐HSD and, unlike endogenous glucocorticoids, they do not bind to corticosteroid binding globulin (Murphy et al., 2007; Singh, Cuffe, & Moritz, 2012). These mechanisms, associated with selective binding of exogenous glucocorticoids to GR, exacerbate the effects of exogenous corticosteroid administration on fetal development.

In addition to corticosteroids, other hormones such as catecholamines, vasopressin, and oxytocin are released upon stress situations and may affect brain development and function (Vargas‐Martínez, Uvnäs‐Moberg, Petersson, Olausson, & Jiménez‐Estrada, 2014; Wyrwoll & Holmes, 2012). However, evidence is still scarce concerning their impact on placental function and fetal development. It is plausible that these substances may have a role in susceptibility to later disease by modifying placental and fetal 11β‐HSD levels, nutrient transport, or GR expression.

### 3.2 Maternal HPA axis dysfunction and fetal and postnatal neurodevelopment

Exposure to high levels of stress during pregnancy is associated with elevated maternal and fetal plasma corticosteroid levels (Hompes et al., 2012). Fetal corticosteroid levels may rise as a result of direct maternal transfer across the placenta, maternal CRH stimulation of fetal HPA axis, or as a consequence of maternal glucocorticoid‐stimulated placental CRH production, which activates fetal HPA axis. Evidence in humans and animals indicates that intrauterine exposure to stress or its glucocorticoid hormone mediators, endogenous or synthetic, has negative impact on brain development (Buss et al., 2012; Pryce et al., 2005; Pryce, 2008), although individual brain areas may only be vulnerable after certain threshold (magnitude or duration of hypercortisolism) has been reached. A “fetal programming” effect, with resetting of HPA axis, is thought to underlie these effects (Moisiadis & Matthews, 2014a, b; Waffarn & Davis, 2012). Long‐lasting deficits in cognitive, affective, as well as addictive behaviors have been associated with prenatal contact to maternal stress and glucocorticoids excess. Schizophrenia, attention‐deficit/hyperactivity disorder, antisocial behavior, increased vulnerability to post‐traumatic stress disorder, anxiety disorders, learning difficulties, and depression were already reported to have an association with prenatal glucocorticoid excess (Barbazanges, Piazza, Le Moal, & Maccari, 1996; Buss et al., 2012; Chrousos & Kino, 2009; Wyrwoll & Holmes, 2012).

Several animal studies have focused these prenatal glucocorticoid‐induced behavioral deficits. In utero glucocorticoid exposure in rats induced pronounced anhedonic behavior and impairment in social interaction in both juvenile and adult animals (Borges et al., 2013, b). High‐dose antenatal corticotherapy also induces deficits in fear memory, triggers anxiety‐like behavior, increases drug‐seeking behavior, and impairs the animal's resilience to stress in adulthood (Oliveira et al., 2006, 2012; Rodrigues et al., 2012). In contrast to synthetic glucocorticoids, milder phenotypes were seen in the natural glucocorticoid group, probably due to inactivation of corticosterone in the placenta (Oliveira et al., 2006). These long‐lasting emotional and social behaviors were associated with a profound reduction in mesolimbic dopaminergic transmission (Borges, Coimbra, Soares‐Cunha, Miguel Pêgo et al., 2013; Borges, Coimbra, Soares‐Cunha, Ventura‐Silva et al., 2013). The behavioral deficits and reduced dopamine levels in nucleus accumbens and amygdala were both reversed by L‐DOPA administration (Rodrigues et al., 2012). Some of these behavioral traits were correlated with neuroanatomical changes. Particularly, fear conditioning and hyperanxiety were associated with increased volume of the bed nucleus of the stria terminalis due to increased dendritic length while opposite effects were seen in the amygdala that presented reduced volume due to significant dendritic atrophy (Oliveira et al., 2012). Leão et al. and Rodrigues et al. had already reported neuroanatomical changes in rats exposed to dexamethasone during late gestation, such as significant reduced volume and cell number in the nucleus accumbens, as well as impoverished dopaminergic innervation of this limbic structure by the ventral tegmental area (Leão et al., 2007; Rodrigues et al., 2012). Altogether, these findings suggest a close link and interplay between glucocorticoids/stress, impaired behavior, and dopaminergic tone. In fact, some of the behavioral disorders that have been associated with prenatal stress or manipulation of the maternal glucocorticoid milieu are related to dopaminergic transmission in the mesolimbic, mesocortical, and nigrostriatal systems (e.g., schizophrenia, drug addiction, and, possibly, depression); (Rodrigues et al., 2012). Prenatal glucocorticoid exposure was also found to modulate long‐term activity of other neural pathways, particularly the mesopontine cholinergic pathway which was implicated in anxious behavior and enhanced stress reactivity by modulating HPA axis function (Borges et al., 2013, b). Behavior studies were also performed in primates. Particularly, repeated betamethasone administration to pregnant baboons resulted in impaired learning and attention disorders in 3‐year‐old female offspring (Rodriguez et al., 2011).

The molecular mechanisms underlying the resetting of HPA axis, after endogenous or synthetic prenatal glucocorticoid exposure, have also been extensively studied. Clear key roles of epigenetic changes in CRH, intracellular GR and MR, 11β‐HSD1, and 11β‐HSD2 have been established (Avishai‐Eliner, Eghbal‐Ahmadi, Tabachnik, Brunson, & Baram, 2001; Diaz et al., 1998; Waffarn & Davis, 2012). Some of the epigenetic alterations were also found to occur in primordial germ cell formation, which can persist through fertilization and development of the subsequent generation (Cottrell & Seckl, 2009; Moisiadis & Matthews, 2014a, b).

Chronic glucocorticoid excess upregulates 11β‐HSD1 in the hippocampus and peripheral metabolic organs increasing local and systemic glucocorticoid levels in rodents (Shoener, Baig, & Page, 2006). Additionally, evidence in rat and human trophoblasts shows that maternal undernutrition or other types of stress that culminate in hypoxia, high catecholamines, and inflammatory cytokines levels can paradoxically downregulate placental 11β‐HSD2, exposing the fetus to excessive amounts of glucocorticoids (Chisaka, Johnstone, Premyslova, Manduch, & Challis, 2005; Homan, Guan, Hardy, Gratton, & Yang, 2006; Mairesse et al., 2007). Differential expression levels of the 11β‐HSD2 enzyme correlate with different patterns of methylation of the promoter and coding regions of this gene in human cells in vitro and in rats in vivo (Alikhani‐Koopaei, Fouladkou, Frey, & Frey, 2004).

Studies in rodents show that prefrontal cortex and limbic regions, such as hippocampus and amygdala, seem to be particularly sensitive to exposure to stress or high levels of glucocorticoids by a mechanism dependent on GR expression, which mediate most of the detrimental effects of glucocorticoids (Buss et al., 2012; Diaz Heijtz, Fuchs, Feldon, Pryce, & Forssberg, 2010). As an example, deficiency of placental 11β‐HSD2 reduces hippocampal GR expression, while increases amygdala GR mRNA levels (Welberg, Seckl, & Holmes, 2000). Low hippocampal GR seem to reduce glucocorticoid negative feedback and lead to exaggerated HPA responses to stress and an increased anxiety‐like behavior in adulthood. On the other hand, increased GR expression in the amygdala is associated with an anxiogenic phenotype (Welberg et al., 2000). Conversely, high levels of maternal care lead to increased GR mRNA expression in the hippocampus and prefrontal cortex and reduced HPA axis responses, resulting in lower plasma glucocorticoid levels and a less anxious phenotype as adults (Liu et al., 1997). In fact, postnatal events, such as maternal care and handling, can probably reverse the prenatal stress‐induced low levels of hippocampal corticosteroid receptors through epigenetic mechanisms that involve thyroid hormone release and activation of serotoninergic pathways to the hippocampal region of the brain, restoring GR expression and recovery of HPA function (Meaney, Aitken, & Sapolsky, 1987; Weaver et al., 2007). These alterations in postnatal brain corticosteroid receptor expression patterns and behavior phenotype support the use of postnatal interventions to reverse modified HPA activity induced by prenatal exposure to endogenous or synthetic glucocorticoids.

CRH has also been appointed as a direct mediator of early‐life stress on later cognitive and behavioral outcomes. High concentrations of CRH are found in situations of maternal stress and growth‐retarded fetuses (Weinstock et al., 1992; Weinstock, 2005). Handling of neonatal rat pups reduced hypothalamic CRH expression and reduced stress‐induced glucocorticoid release, enhancing hippocampal GR expression in adult animals, in a well‐defined sequence (Avishai‐Eliner et al., 2001).

Besides molecular alterations in key elements of HPA axis, remarkable brain structural changes have been described after in utero exposure to elevated levels of glucocorticoids, as already discussed above. Evidence on rodents and nonhuman primates showed that prenatal stress, excess exogenous glucocorticoids, and inhibition of 11β‐HSD2 have influence on fetal neurogenesis and hippocampal anatomy (Lemaire, Koehl, Le Moal, & Abrous, 2000; Schmitz et al., 2002). Coe et al. (2003) demonstrated that moderate stress, both early and late in rhesus monkey pregnancy, resulted in a 10%–12% decrease in hippocampal volume, still not recovered by 2 years postpartum. They also have described an inhibition of neurogenesis in the dentate gyrus and disturbed size and shape of the corpus callosum. Administration of single and repeated courses of corticosteroids to pregnant sheep and rhesus monkeys retarded fetal brain growth and was followed by a reduction in brain weight at term that persisted into adulthood (Huang et al., 1999; Moss et al., 2005; Uno et al., 1990). These findings are highly relevant as they provide evidence for a brain structure–function relationship.

The consequences of prenatal stress/glucocorticoid exposure in humans are not so well known; however, evidence suggests some overlapping findings with preclinical studies. Higher maternal cortisol levels in earlier human gestation were associated with a larger right amygdala volume and more affective problems in girls, including anxious behavior and exaggerated stress reactivity (Buss et al., 2012). Children from mothers who self‐report high levels of stress/anxiety during pregnancy (associated with high salivary cortisol levels) were described to have higher basal HPA axis activity and self‐reported anxiety at 6 months, 5 years, and 10 years of age, as well as behavioral problems and impaired attention and concentration as toddlers (O'Connor et al., 2005; Talge, Neal, & Glover, 2007). Moreover, in human pregnancies complicated by intrauterine growth restriction, fetal cortisol levels are elevated at term, associating reduced fetal growth rates with elevated glucocorticoids (Goland et al., 1993). The long‐term effects of prenatal stress and glucocorticoid excess during pregnancy depend on timing of exposure as well as on the sex of the offspring (Brunton & Russell, 2011; Davis & Sandman, 2010). In fact, many aspects of adverse fetal programming affect more males than females (Brunton & Russell, 2010; Dunn, Morgan, & Bale, 2011). In humans, placenta of female fetuses may convey a relative protection from glucocorticoid excess due to increased glucocorticoid inactivation by feto‐placental 11β‐HSD2 (Clifton & Murphy, 2004).

After Liggins and Howie published the first randomized controlled trial on the effect of exogenous glucocorticoids on human fetal neurodevelopment, several studies have also been performed aiming to clarify the safety of antenatal corticosteroid administration to pregnant women at risk of preterm delivery (ACOG, 2016; NIH Consensus Statement, 2000; RCOG, 2010). Until some years ago, the use of repeated courses of antenatal corticosteroids was widespread (NIH Consensus Statement, 2000; Zephyrin et al., 2013). The detrimental effects of corticosteroids seem to be dose‐associated, and multiple courses induce more severe damage than single injections of the same total dose (Uno et al., 1990). These facts raise the question whether chronic although low level stress is more detrimental than an acute sharp trauma to fetal development. The main issue of debate concerning antenatal corticosteroid therapy is the long‐term effects in the central nervous system. Nevertheless, long‐term developmental follow‐up studies in infants exposed to repeated doses of prenatal corticosteroids are limited to date and have produced conflicting results.

In humans, Multiple Courses of Antenatal Corticosteroids for Preterm Birth Study (MACS trial) showed that neonates who received multiple courses of corticosteroids have significantly lower mean birthweight, length, and head circumference than those in the placebo group, despite no major improvement in neonatal outcome (Murphy et al., 2008). Other studies found similar results (Abbasi et al., 2000; French, Hagan, Evans, Godfrey, & Newnham, 1999). Repeated antenatal betamethasone injections were associated with a reduced cortex convolutions index and brain surface area in human offspring (Modi et al., 2001). These effects appear to be more pronounced if corticosteroids are administered during late gestation when growth rate is higher and thus most susceptible to the catabolic effects of steroids (Bloom, Sheffield, McIntire, & Leveno, 2001; French et al., 1999). Follow‐up studies in 5‐year‐old children at MACS trial who were exposed to repeated courses of antenatal corticosteroids and born ≥37 weeks showed an increased risk of neurodevelopmental/neurosensory impairment (Asztalos et al., 2013, 2014), while other nonrandomized studies have shown no difference between exposed and nonexposed children (Doyle, Kitchen, Ford, Rickards, & Kelly, 1989; Hasbargen, Reber, Versmold, & Schulze, 2001; MacArthur, Howie, Dezoete, & Elkins, 1982; Thorp et al., 2002).

A 2015 Cochrane Review concluded that, although repeated doses reduced the severity of neonatal lung disease, there were insufficient data to exclude other beneficial or harmful effects to the mother or infant. Although repeated doses were associated with a small reduction in size at birth, this was not significant when adjusted for gestational age. No long‐term benefits or harms were seen at 18 months to 2 years' corrected age although only betamethasone was evaluated (Crowther, McKinlay, Middleton, & Harding, 2015).

There are few follow‐up studies on single course of antenatal corticosteroids exposed fetuses into childhood and adulthood but results have been reassuring. (Collaborative Group on Antenatal Steroid Therapy, 1984; Dalziel et al., 2005; Dessens, Haas, & Koppe, 2000) Just one small study reported neurodevelopmental delay in childhood (Salokorpi et al., 1997) No major long‐term adverse effects have been found on psychomotor, cognitive or neurological development, as well as on working memory, attention, or psychiatric morbidity (Dalziel et al., 2005; Liggins & Howie, 1972). These results reduced previous concerns regarding decreased brain growth after antenatal corticosteroid exposure from animal studies (Huang et al., 1999). Nevertheless, a recent Cochrane Review concluded that children with and without treatment had similar results for behavioral/learning difficulties and intellectual impairment; also, data were inconclusive about neurodevelopmental delay in childhood and educational achievement and intellectual impairment in adulthood (Roberts, Brown, Medley, & Dalziel, 2017). Further follow‐up studies in neurodevelopmental effects in later childhood and adulthood of single and repeated courses of antenatal corticosteroids are needed.

Concerning neonatal corticosteroid treatment, some deleterious effects were reported. Particularly, cerebral cortical gray matter volume in premature infants treated with dexamethasone was reduced by 35% when compared to nontreated infants, as well as a significant decrease in cerebellum volume (Modi et al., 2001; Murphy et al., 2001; Tam et al., 2011). Other regions of the brain can also be affected (Antonow‐Schlorke et al., 2009; Murmu et al., 2006).

### 3.3 Treatment options and guidelines

Antenatal corticosteroid therapy administered to women at risk of preterm delivery has brought a significant decrease in the incidence of respiratory distress syndrome (RDS), intraventricular hemorrhage (IVH), necrotizing enterocolitis, and overall neonatal mortality (ACOG, 2016; NIH Consensus Statement, 2000; Royal College of Obstetricians and Gynaecologists, 2010). The most extensively studied corticosteroids for the prevention of RDS, since Liggins initial clinical studies with sheep, are betamethasone and dexamethasone (Liggins, Schellenberg, Manzai, Kitterman, & Lee, 1988; Schellenberg, Liggins, Manzai, Kitterman, & Lee, 1988). However, fetal exposure to other synthetic glucocorticoids may occur in the setting of a variety of medical conditions, including asthma and autoimmune disorders. Additionally, association of thyroid hormones or TRH to prenatal corticosteroids has been suggested to further reduce RDS and neonatal lung disease in preterm infants (Liggins et al., 1988; Schellenberg et al., 1988). Nevertheless, a Cochrane systematic review from 2013 showed that prenatal TRH in addition to corticosteroids, given to women at risk of preterm birth, does not improve infant outcomes and is associated with maternal side effects (Crowther, Alfirevic, Han, & Haslam, 2013).

At the moment, major organizations such as National Institutes of Health (NIH), American College of Obstetricians and Gynecologists (ACOG), and Royal College of Obstetricians and Gynaecologists (RCOG) recommend a single course of antenatal corticosteroid treatment for pregnant women between 24 and 34 weeks of gestation at risk of preterm delivery within the next 7 days (ACOG, 2016; Hofmeyr, 2009; NIH Consensus Statement, 2000; Roberts et al., 2017; Royal College of Obstetricians and Gynaecologists, 2010). A single rescue course of antenatal corticosteroids should be considered if gestational age is less than 34 0/7 weeks, delivery is likely to occur in the next 7 days, and the antecedent treatment was given more than 2 weeks before (ACOG, 2016). Recently, ACOG published a Practice Advisory, endorsed by American Academy of Pediatrics, based on findings of Antenatal Late Preterm Steroids (ALPS) trial demonstrating that administration of antenatal betamethasone may be of benefit for pregnancies, not previously exposed to antenatal corticosteroids, at high risk of late preterm birth between 34 0/7 and 36 6/7 weeks of gestation (ACOG, 2016; Saccone & Berghella, 2016). However, neurodevelopment outcomes were not addressed by ALPS trial.

The currently approved regimen doses for betamethasone and dexamethasone were first selected arbitrarily, but pharmacodynamic studies suggested that both regimens resulted in about 75%–80% occupancy of available corticosteroid receptors, providing near maximal corticosteroid receptor‐mediated response in fetal target tissues (Ballard, Granberg, & Ballard, 1975). Higher and more frequent doses were not shown to improve perinatal benefits and, indeed, increased the likelihood of adverse effects (NIH Consensus Statement, 2000). Nevertheless, despite effectiveness in reducing most morbidities and mortality related to prematurity, there is insufficient evidence on which to base a strong recommendation for use of one drug over the other, as well as optimal dose, timing, frequency, and route of administration (Jobe & Soll, 2004). In a systematic review, Brownfoot, Gagliardi, Bain, Middleton, and Crowther (2013) concluded that dexamethasone may have some benefits compared with betamethasone, such as less intraventricular hemorrhage and periventricular leukomalacia and a shorter length of stay in the neonatal intensive care unit. Some concerns have been raised about dexamethasone risk of poorer motor skills and coordination, lower IQ scores, and an increased frequency of clinically significant disabilities in survivors (Brownfoot, Crowther, & Middleton, 2008; Lee, Stoll, McDonald, & Higgins, 2006; Yeh et al., 2004).

In conclusion, intrauterine exposure to high levels of synthetic or even endogenous glucocorticoids seems to have a definitive impact on central nervous system development with long‐term susceptibility to cognitive, behavior, and affective disorders. The increased reactivity of the HPA axis is thought to underlie these manifestations, although the exact molecular mechanisms which explain these outcomes need further investigation (Table 4). As far as possible, the developing brain should be protected against the effects of pre‐ and postnatal stress.

**Table 4:** Glucocorticoid hormones: Issues on debate

Understand the molecular mechanisms by which glucocorticoids affect fetal neurodevelopment
Determine the relationship between maternal stress/anxiety during pregnancy and fetal cortisol levels
Understand the differential impact on fetal brain growth of self‐reported maternal stress/anxiety or high maternal cortisol levels during pregnancy
Establish if the influence of high cortisol levels on fetal brain growth translates into adverse neurological outcomes in later life
Establish the long‐term effects in the central nervous system of patients who undergone single and rescue courses of antenatal corticosteroids
Determine which exogenous glucocorticoids have the best safety profile for antenatal administration

## 4 CROSS‐REGULATION OF THE THYROID HORMONE AND GLUCOCORTICOIDS

Although much attention has been driven to the effects of stress‐related HPA axis mechanisms on fetal neurodevelopment, hypothalamus–pituitary–thyroid (HPT) axis is also a stress‐sensitive system and both axes interact with each other during fetal development (Moog et al., 2017). Particularly, both thyroid hormones and glucocorticoids mediate several maturational effects, essential for neonatal survival, acting synergistically to switch cell cycle from proliferation to differentiation (Bernal, 2017; Harris & Seckl, 2011; Liggins et al., 1988).

In human adults, glucocorticoids generally inhibit thyroid function. In fact, glucocorticoid administration was shown to decrease plasma TSH levels, as well as TSH response to TRH stimulation (Ahlquist, Franklyn, Ramsden, & Sheppard, 1989; Taylor, Flower, & Buckingham, 1995). Additionally, depression or chronically increased cortisol levels were associated with lower levels of cerebral thyroid hormones, probably due to a cortisol‐related decrease in brain deiodinase 2 activity. Notwithstanding, acute stress was shown to induce synthesis and secretion of thyroid hormones, apparently by a mechanism involving glucocorticoids (Hidal & Kaplan, 1988; Jackson, 1998; Nadolnik, 2012). Similarly, during fetal development, at least in sheep models, maternal and fetal administration of glucocorticoids, during third trimester, increased plasma T3 but not T4 levels, perhaps secondary to modifications in fetal thyroid hormones metabolism, namely a decrease in placental deiodinase 3 activity (Forhead et al., 2007; Thomas, Krane, & Nathanielsz, 1978). In fetal central nervous system, the stimulatory role of glucocorticoids in thyroid hormone metabolism may be accomplished by means of iodine metabolism modulation, influence on deiodinases activity, and thyroid hormone receptor expression (Nadolnik, 2012). Whether the effect of glucocorticoids on fetal T3 levels is the same in early pregnancy, where fetal thyroid hormones are more dependent on maternal fraction and higher expression levels of placental deiodinase 3 are observed, needs further investigation.

Maternal stress, by activation of HPA axis, may play a role in premature maturation of fetal HPT axis and other fetal tissues, by means of an increase in fetal T3 concentrations (Slone‐Wilcoxon & Redei, 2004). Nevertheless, regarding fetal neurodevelopment, in the presence of maternal stress and/or chronically increased cortisol levels, despite the increase in fetal T3 concentrations, maternal thyroid hormones most likely decrease, which means that lower levels of maternal T4 cross placental barrier and reach fetal brain, compromising fetal neurodevelopment. Additionally, low maternal thyroid hormones impair renal cortisol clearance and decrease its metabolism into inactive cortisone, apparently by decreasing 11β‐HSD2 activity. Both low T3 and high glucocorticoids in fetal brain have a deleterious impact on fetal neurodevelopment, most likely in brain regions particularly vulnerable to both hormones, such as hippocampus (Gould, Woolley, & McEwen, 1991; Hellstrom, Dhir, Diorio, & Meaney, 2012).

On the other hand, administration of thyroid hormones to neonatal rat pups stimulates CRH, ACTH, and glucocorticoid secretion, as well as hippocampal GR concentration, which modulates future HPA axis response to stress by enhancing glucocorticoid negative feedback sensitivity (Johnson et al., 2013; Meaney et al., 1987; Shi, Levy, & Lightman, 1994). Furthermore, postnatal modulators, such as maternal care, can regulate HPA axis responses by means of T3 release and increased conversion of T4 to T3 (Hellstrom et al., 2012). Thus, high levels of thyroid hormones also accelerate HPA axis maturation.

In conclusion, disruption of either HPA or HPT axes during fetal neurodevelopment can permanently program the other axis with lifelong deleterious consequences in terms of susceptibility for neurodevelopmental diseases.

## 5 OTHER MATERNAL HORMONAL AXIS INFLUENCE ON FETAL NEURODEVELOPMENT

Regardless of thyroid hormones and glucocorticoid influence in fetal brain growth, several other hormones have been pointed out to have a role in fetal neurodevelopment. Scientific evidence concerning other maternal hormonal axes is still limited and requires further extended and validated studies. Nevertheless, promising findings address the effect of some maternal hormonal axes on fetal neurogenesis and later physiologic responses related to stress adaptation and emotion regulation, sometimes by interfering with the HPA axis. Herein, we highlight some evidence concerning sex steroids, oxytocin, and melatonin.

The developing brain is vulnerable to the action of sex steroids. Studies of the organizing effects of gonadal steroids on brain structure and behavior have been performed mainly in rodents. Concerning reproductive behavior, sexual preference of a male for a female is likely to be controlled by the sexually dimorphic nucleus of the preoptic area which is differentially shaped in male and female animals by the action of testosterone during late embryonic life and the first days of postnatal life (Balthazart, 2011). Besides sexual behavior, both fetal androgens and estrogens have also been associated with differential growth in other sexually dimorphic brain areas, including prefrontal cortex, cerebellum, amygdala, and hippocampus (Neufang, Specht, & Hausmann, 2009; Peper et al., 2009), explaining sex differences found in cognitive (Berman et al., 1997) and affective skills later in life (Amin, Epperson, Constable, & Canli, 2006; Lombardo et al., 2012). Other factors contribute to sex differences in brain morphology including enzymes involved in sex steroids biosynthesis and metabolism, such as aromatase (Biegon et al., 2010). Proteins encoded on sex chromosomes and environmental factors are also involved (McCarthy, Arnold, & Ball, 2012). Sex steroids actions may be accomplished via classical genomic receptors, as well as nonclassical membrane‐associated receptors. In developing humans and rodents, sexually dimorphic brain areas were found to express high density of sex steroid receptors (Takeyama et al., 2001). Specific structural effects of prenatal androgens, estrogens, and progesterone are neurite outgrowth and synaptogenesis, dendritic branching, and myelination (Garcia‐Segura & Melcangi, 2006; Haraguchi et al., 2012). The precise molecular mechanisms for these hormonal effects are still being elucidated but include activation of programmed cell death, differential expression of transcription factors and microRNAs, DNA methylation, and histone post‐translational modifications of genes, including steroid receptors genes (Gore, Martien, Gagnidze, & Pfaff, 2014). Interactions between sex hormones and neurotransmitters, such as serotonin, dopamine, GABA, and glutamate have also been described to be of relevance in animals and humans (Barth, Villringer, & Sacher, 2015).

Disruptions in maternal sex steroids production and metabolism, at critical stages in development, might influence normal fetal brain structure and functional outcomes, including permanent changes in non‐reproductive behavior (Weiss, 2002). It is still controversial whether or not endogenous maternal sex steroids within physiologic range of pregnancy have an effect on fetal hormone levels. In animals, prenatal treatment of rats with estradiol or testosterone resulted in sexually dimorphic changes in social play behavior and performance in memory tests, as well as alteration in the size of hippocampal pyramidal cells (Auger & Olesen, 2009; Isgor & Sengelaub, 1998). Similarly, developmental exposure to estrogenic endocrine disruptors in rodents was associated with sexually dimorphic social and anxiety behaviors and learning difficulties in adolescence and adulthood (Carbone et al., 2013; Kundakovic et al., 2013) Additionally, fluctuations on progestin levels in pregnant rats resulted in significant differences in performance on hippocampal‐dependent tasks in the offspring (Paris, Brunton, Russell, Walf, & Frye, 2011). In humans, there were observed sexually dimorphic effects of sex steroids on behavior, with increased cord sex hormones (androgens, estrogens and progesterone) affecting mood and cognition (Jacklin, Wilcox, & Maccoby, 1988; Marcus, Maccoby, Jacklin, & Doering, 1985). Increased levels of sex hormones during fetal development might also predispose male children to autism spectrum disorders (Malkki, 2014).

Insight into human genetic disorders, pathophysiological conditions, and pharmaceutical treatments, which result in changes in the hormonal milieu of the mother and developing fetus, has also provided some knowledge about the role of prenatal sex steroids on fetal neurodevelopment (Gore et al., 2014). For example, congenital adrenal hypertrophy, characterized by increased prenatal production of adrenal progestins and androgens, was associated with more masculinized and autistic behavioral and cognitive traits in girls (Knickmeyer et al., 2006). In offspring of mothers with polycystic ovary syndrome, also characterized with elevated maternal androgens, higher testosterone levels in the amniotic fluid were associated with an autistic phenotype, with a larger impact on females compared with males (Palomba et al., 2012). Maternal functional polymorphisms in the sex steroid synthesis and metabolism pathways that are associated with higher estrogen levels were related to attention problems, hyperactivity, and poorer adaptive skills in male offspring (Miodovnik et al., 2012) Furthermore, studies on behavioral outcomes after prenatal exogenous sex steroid exposure in pregnancies at risk of early fetal loss and premature birth showed significant differences in personality and behavior between groups, albeit without criteria for any disorder and with little effects on cognition (Reinisch, 1977). A slight, although nonsignificant, association was also established between ovulation‐inducing drugs and autism spectrum disorders (Hvidtjørn et al., 2011).

There seems to exist a cross talk between fetal glucocorticoids and sex steroid hormones. In fact, prenatal stress, by stimulating HPA axis response which, in turn, upregulates adrenal androgen activity, was also shown to promote a sexually dimorphic response in animal models (Barrett & Swan, 2015; Hill et al., 2014; Mueller & Bale, 2008) These effects, more consistently seen in developing males, include reproductive anomalies (Van den Driesche et al., 2011), feminization of play behavior, and anxiety‐related behaviors that are concomitant with changes in sexually dimorphic brain nuclei (Arnold & Gorski, 1984). In humans, investigation shows that prenatal stress is associated with several disorders with sex differences in prevalence such as autism spectrum disorders, schizophrenia, and attention‐deficit/hyperactivity disorder (Khashan et al., 2008; Kinney, Munir, Crowley, & Miller, 2008).

Additionally, the neuropeptide oxytocin may also be one of the interveners of the fetal programming effect (Carter, 2014; Freedman, Brown, Shen, & Schaefer, 2015; Kenkel, Yee, & Carter, 2014). While endogenous oxytocin has neuroprotective effects during labor, excessive exogenous oxytocin increases the risk of fetal hypoxic–ischemic events (Ben‐Ari, Khalilov, Kahle, & Cherubini, 2012; Ceanga, Spataru, & Zagrean, 2010; Khazipov, Tyzio, & Ben‐Ari, 2008). Besides that, evidence in humans and animals suggests that perinatal exogenous oxytocin may be associated with later cognitive impairment and affective and mood disorders (Li, Gonzalez, & Zhang, 2012; Lucht et al., 2009; Scantamburlo et al., 2007), including attention‐deficit/hyperactivity disorder, bipolar disease, as well as autism (Gregory, Anthopolos, Osgood, Grotegut, & Miranda, 2013; Kurth & Haussmann, 2011). In both rodents and humans, oxytocin can influence emotionality and/or social behaviors by promoting downregulation of the HPA axis, through ACTH inhibition (Vargas‐Martínez et al., 2014). Leuner, Caponiti, and Gould (2012) found that oxytocin stimulates both cell proliferation and adult neurogenesis in the ventral portion of the hippocampal dentate gyrus of rats, even in those treated with glucocorticoids or exposed to a stressor. These findings suggest that oxytocin may protect against the suppressive effects of stress hormones on hippocampal plasticity.

Furthermore, melatonin is a neuroendocrine hormone secreted by pineal gland and generated under the control of an endogenous circadian clock in the suprachiasmatic nucleus of the hypothalamus (Mauriz, Collado, Veneroso, Reiter, & González‐Gallego, 2013; Radogna, Diederich, & Ghibelli, 2010). Abnormal patterns and/or reduced levels of maternal melatonin secretion may be associated with obstetric complications such as neonatal neurological disability, through mechanisms of epigenetic modifications (Tamura et al., 2008). Melatonin also seems to affect the circadian plasma concentrations of other critical hormones during gestation, such as prolactin and cortisol (Patrick et al., 1980). The disruption of the circadian melatonin rhythm impairs neurogenesis in rats and is thought to interrupt REM sleep in developing animals, resulting in diminished brain growth, as neuronal activation occurs mainly during this sleep period (Guzman‐Marin et al., 2005; Morrissey, Duntley, Anch, & Nonneman, 2004). Melatonin also appears to be neuroprotective because maternally administered melatonin prevents oxidative lipid and DNA and mitochondrial damage in the brain of mature and premature fetal rats (Wakatsuki et al., 2001).

## 6 CONCLUSION

The maternal hormonal milieu can provide the ideal or deleterious conditions for several aspects of fetal development, and, particularly, fetal brain development is one point of great concern for researchers and clinicians. In this context, thyroid hormones and cortisol have been the most frequently studied hormones over the last few years; nevertheless, there are several other hormones that seem to influence fetal neurodevelopment. Additionally, it appears to exist a considerable cross talk between different hormonal axes which is still poorly understood. Notably, their impact on intrauterine central nervous system development persists throughout life and may be the cause of impaired neurodevelopment, including cognitive, behavior, and affective disorders later in life. For most of these hormones, there are no established reference values for pregnancy, or even for nonpregnant population. For others, such as thyroid hormones, poorer neurodevelopment outcomes have been described for maternal circulating levels that are still within the normal reference range for adults. In conclusion, investigation should proceed in order to improve fetal neurodevelopment outcome associated with maternal hormonal milieu.

## CONFLICT OF INTEREST

We wish to confirm that there is no known conflict of interests associated with this publication and there has been no significant financial support for this work that could have influenced its outcome. We confirm that the manuscript has been read and approved by all named authors and that there are no other persons who satisfied the criteria for authorship but are not listed. We further confirm that the order of authors listed in the manuscript has been approved by both of us. We confirm that we have given due consideration to the protection of intellectual property associated with this work and that there are no impediments to publication, including the timing of publication, with respect to intellectual property. In so doing, we confirm that we have followed the regulations of our institutions concerning intellectual property.

## AUTHOR CONTRIBUTIONS

AM provided substantial contribution to the conception and drafting of the review; NS had an important contribution in critically revising the work for important intellectual content and also gave the final approval of the version to be published.
